# Empirically derived dietary patterns and postpartum depression symptoms in a large sample of Iranian women

**DOI:** 10.1186/s12888-023-04910-w

**Published:** 2023-06-13

**Authors:** Shima Dehghan-Banadaki, Mahdieh Hosseinzadeh, Farzan Madadizadeh, Hassan Mozaffari-Khosravi

**Affiliations:** 1 International Campus, Shahid Sadoughi University of Medical Sciences, Yazd, Islamic Republic of Iran; 2 Department of Nutrition, School of Public Health, Shahid Sadoughi University of Medical Sciences, Yazd, Islamic Republic of Iran; 3 Department of Biostatistics and Epidemiology, School of Public Health, Shahid Sadoughi University of Medical Sciences, Yazd, Islamic Republic of Iran

**Keywords:** Postpartum Depression, Dietary patterns, Maternal and child health cohort study, Yazd

## Abstract

**Background:**

Postpartum Depression (PPD) is a major depressive disorder that mainly begins within one month after delivery. The present study aimed to determine the relationship between dietary patterns and the occurrence of high PPD symptoms in women participating in the initial phase of the Maternal and Child Health cohort study, Yazd, Iran.

**Methods:**

This cross-sectional study was carried out in the years 2017–2019 included 1028 women after childbirth The Food Frequency Questionnaire (FFQ) and the Edinburgh Postnatal Depression Scale (EPDS) were study tools. The EPDS questionnaire was used to measure postpartum depression symptoms and a cut-off score of 13 was considered to indicate high PPD symptoms. The baseline data related to dietary intake was collected at the beginning of the study at the first visit after pregnancy diagnosis and the data related to depression, were collected in the second month after delivery. Dietary patterns were extracted by exploratory factor analysis (EFA). Frequency (percentage) and mean (SD) were used for description. Chi-square test, Fisher’s exact test, independent sample t-test, and multiple logistic regression (MLR) were used for data analysis.

**Results:**

The incidence of high PPD symptoms was 24%. Four posterior patterns were extracted including prudent pattern, sweet and dessert pattern, junk food pattern and western pattern. A high adherence to the western pattern was associated with a higher risk of high PPD symptoms than a low adherence (OR_T3/T1_: 2.67; p < 0.001). A high adherence to the Prudent pattern was associated with a lower risk of high PPD symptoms than a low adherence (OR_T3/T1_: 0.55; p = 0.001). There are not any significant association between sweet and dessert and junk food patterns and high PPD symptoms risk (p > 0.05).

**Conclusion:**

High adherence to prudent patterns was characterized by high intake of vegetables, fruit and juice, nuts and beans, low-fat dairy products, liquid oil, olive, eggs, fish, whole grains had a protective effect against high PPD symptoms, but the effect of western pattern was characterized by high intake of red and processed meats and organs was reverse. Therefore, it is suggested that health care providers have a particular emphasis on the healthy food patterns such as the prudent pattern.

## Introduction

Depression is one of main causes of disease burden worldwide, and almost one in five people experiencing a major depressive disorder during their lifetime. Over the course of a lifetime, depression is almost twice as common in women as in men [1] and it is especially common during and in the first year after giving birth. The antepartum and postpartum periods of time may be important times to diagnosis and decrease the risk of depression [2]. The formation of the fetus and its birth cause mental health disorders including depression [3].

Pregnancy creates various hormonal, physical, emotional and psychological changes in women, so that different emotions from joy and pleasure to sadness and crying arise in the mother [4, 5]. “Baby blues” is a kind of feeling of sadness and discomfort that occurs after birth of a child which decreases in the first 2 weeks after delivery in mothers [3, 5–9] but postpartum depression (PPD) occurs up to one month after childbirth which has more severe and long lasting effects on mothers [10]. PPD threatens the health of the mother and her baby, and a depressed mother has the possibility of harming herself and her child, also it affects the health of the entire family, including husband and other family members [11, 12]. PPD can be the beginning of other and longer-term disorders and untreated depression can have a direct impact on a woman’s emotional life by causing despair and sadness, sleep disorder, anxiety, irritability, decreased libido. Previous studies have mentioned the long-term effect of PPD and considered it to be the cause of women’s inability to return to normal function and even defined it as a negative factor on the mother-neonatal relationship [13–16] and in some cases, it may be associated with suicidal thoughts and behaviors [17, 18].

The prevalence statistics of PPD disorder among women at the global level indicate a rate of 16.6–18.8%(17.7%) percent [19] Evidence demonstrates that all countries face the challenge of PPD, but low- to middle- income countries face the greatest burden [20–22]. PPD prevalence in Iran was estimated at about 16 to 38% [14]. In a study using Beck depression scale for screening of PPD among 6628 women during 2–12 months after delivery in rural parts of Isfahan province in Iran, the prevalence of moderately and severely depressed women was 19.3% and 19.8%, respectively [23] also, the results of a systematic review and meta-analysis in 2022 showed that during the coronavirus pandemic, the prevalence of PPD in the world increased and was estimated at about 34% with a 95% confidence interval (0.21–0.46) [24].

Although PPD is considered as a major health problem, but all the main reasons for its creation have not been identified. Various studies have identified several risk factors such as history of depression, number of children, occupation, socio-economic status, age, education level, gender of the baby [11, 16, 25–27]. Other studies have introduced the nutritional factor as an influencing factor on depression [28, 29]. In these studies, nutrition has been examined partially based on food units, while considering food patterns can provide better results because the combination of different foods and their interaction can have different effects on depression. Maracy et al. 2014 in their study on the relationship between dietary patterns and PPD, showed that a dietary pattern with high amount of fruits and vegetables can reduce the risk of postpartum depression [30]. Chatzi et al. showed that following a diet containing fruits, vegetables, dairy products, olives, fish and nuts can reduce postpartum depression [31]. While, there were studies that pointed out the absence of a relationship between dietary patterns and the risk of depression [32].

According to the literature review, there were contradictory results on the relationship between dietary patterns and depression. Also, different regions of the world have their own specific dietary patterns and diseases, so to determine the role of dietary patterns in PPD, it seems necessary to conduct other studies in different regions of the world, moreover with the rapid conversion of social economy status, the diversity of food has increased, so the interactions between various foods and nutrients have become more complicated. Therefore, the present study aimed to determine the relationship between dietary patterns and the occurrence of high PPD symptoms using posteriori method in women participating in the initial phase of the Maternal and Child Health cohort study, Yazd, Iran.

## Methods

Data were obtained from the Maternal and Child Health cohort study in Iran, started in the fall 2017 until the winter 2019. This study is a part of the cohort study that is being conducted on 3000 women referring for pregnancy care health centers. Women are followed up until the end of pregnancy and also evaluate the baby and the health of the mother two months after giving birth. Among the 3000 mothers included in the cohort, in our study, 1038 mothers were included for postpartum evaluation and completed the questionnaire two months after delivery. Of these mothers, 4 participants due to not completing the FFQ and 3 participants due to reporting total calories intake below 800 or above 6000 and 3 participants due to not completing the EPDS questionnaire were excluded of the study and 1028 mothers were included in this cross-sectional study. 1028 women were included in this cross-sectional study. Pregnant mothers who completed the mental status questionnaire (MSQ) two months after giving birth were included in the study [33], one of the parts of MSQ questionnaire was EPDS, which was used to assess the presence of postpartum depression symptoms. The inclusion criteria was living in Yazd province, which is located in the central part of Iran, completing the informed consent form, intending to stay in Yazd province for at least 4 years, being at least 15 years old, receiving services from health centers in Yazd province during last year, completing food frequency questionnaire (FFQ) [34]at the beginning and during of pregnancy. Exclusion criteria was the presence of mental illness or depression before pregnancy, specific illness including epilepsy and nephropathy or cancer, abnormal pregnancy. All procedures in the study involving human subjects were approved by the Research Ethics Committees at Shahid Sadoughi University of medical sciences, Yazd, Iran.

### Dietary data collection

The dietary intake was assessed by a semi quantitative food frequency questionnaire, included 88 items with specific serving sizes. The FFQ contains the following seven frequency options: almost never, less than once a week, once a week, 2–3 times a week, 4–6 times a week, once a day, and more than two times a day. The participants estimated the portion size, using household measures (cups). The frequency category of each food was calculated to the daily intake. The psychometric properties of this questionnaire in Iran were investigated and confirmed by Sharifi among pregnant female in 2016. In the study of Sharifi et al., Pearson correlation coefficients between test and retest for foods was r = 0.845. The Kaiser-Meyer-Oilskin measure of sampling adequacy was 0.584, P values for the Bartlett test of sphericity were all less than 0.001 [35].

### The edinburgh postnatal depression scale (EPDS)

The postpartum depression was measured by using a questionnaire called the Edinburgh Postnatal Depression Scale (EPDS) which included 10 items of 4-point Likert scale. People who score 13 or higher were considered as high PPD symptoms [21, 36–38]. The psychometric properties of this tool were investigated and confirmed in Iran by Mazhari and Nakhaee in 2007 [39], they considered EPDS of 12/13 as high symptoms of depression score. According to Phillips et al. study, EPDS scores of 13 or over are considered indicative of likely major depression and scores between 10 and 12 of likely minor depression [31, 40]. In the present study we decided to use the cut-off point of 13, as it has been shown to achieve high sensitivity (95%) and specificity (93%) when compared with clinical psychiatric interview [31], and this score is recommended for use when reporting on high depression symptoms in postpartum women.

### Dietary patterns

In measuring the dietary patterns of mothers, according to Khaled et al.‘s 2020 study [41], the items of the FFQ questionnaire were categorized into twenty food groups (Table 1), the similarity of nutrient profiles was the basis of this classification. The food groups are: vegetables, fruit and juice, Nuts and beans, Low-fat dairy products, Liquid oil, olive, egg, fish, whole grains, tea, sweet jam and desserts, refined grain, solid oil, salty snacks, salt, red meat, processed meats and organs, high-fat dairy, Industrial drinks and Birds.

**Table 1:** Food Grouping Used in the Dietary Pattern Analysis

Food Groups	Food Items
Vegetables	types of cabbage, carrots, cooked spinach, lettuce, cucumber, greens, stewed vegetables, eggplant, celery, green peas, green beans, green bell peppers, bell peppers, turnips, pumpkin, stewed squash, mushrooms, raw onions, Fried onions, tomatoes, garlic, potatoes
Fruit and juice	Apple, cherry, apricot, plum, fresh fig, kiwi, strawberry, grape, fresh berry, date, banana, pomegranate, melon, orange, tangerine, grapefruit, pear, persimmon, cantaloupe, melon, watermelon, nectarine, peach and Raisins, dried berries, dried figs, dates and natural juice
Nuts and beans	Almonds, peanuts, walnuts, pistachios, hazelnuts, all kinds of seeds, other nuts and beans, peas, lentils, mung beans, soybeans, beans
Low-fat dairy products	Low-fat milk, normal yogurt, buttermilk
Liquid oil	Flour and liquid oil
olive	olive
egg	egg
fish	All kinds of fish, tuna, fish,
whole grains	Barbari bread, Tanuri bread, Sangak bread and Tufton bread
tea	tea
sweet jam and desserts	Sugar, chocolate, sweet biscuits, honey, sweet jam and desserts
refined grain	Lavash bread, baguette, rice, pasta, and dry bread
solid oil	Butter and animal oil,
salty snacks	Pickles and pickles, chips and puffs,
salt	salt
Red meat	Beef or veal, minced meat, lamb
processed meats and organs	Sausages - breast milk, tongue, brain, head, legs, heart, giblets and liver
high-fat dairy	Full-fat milk curd, full-fat yogurt, cream cheese, cream and cheese, condensed yogurt, ice cream, cheese
Industrial drinks	Industrial soft drinks and juices
Birds and chickens	Chickens

Factor analysis with the principal component factor extraction method was conducted using the statistical software package IBM SPSS Statistics version 23.0, and varimax rotation was used to make uncorrelated factors easier to interpret. The Kaiser–Meyer–Olkin measure of sample adequacy and the Bartlett test of sphericity were used to assess data adequacy for factor analysis. The result of the Kaiser–Meyer–Olkin test was 0·67 and the Bartlett test was significant (P < 0·001), indicating that factor analysis of the data would be useful. The eigenvalue and scree plot were used to decide which factors remained. After evaluating the eigenvalues, the scree plot test and interpretability, the factors with eigenvalues ≥ 1·0 were retained.

### Covariates

A general questionnaire was used to collect information. Well-trained examiners measured the anthropometric indices of participants with light clothing and without shoes. Height was measured to the nearest 0·1 cm and weight was measured to the nearest 0·1 kg. BMI was calculated by dividing weight (in kilograms) by the square of height (in meters). Waist circumference (WC) was measured to the nearest 0·1 cm at the middle point between the lowest point of the rib cage and the iliac crest. The blood pressure was measured in the sitting position after a 10 min rest period. The appearance of the first sound was used to define systolic blood pressure (SBP) and the disappearance of the sound was used to define diastolic blood pressure (DBP). The SBP and DBP were recorded twice, and the average of each measurement was used for data analysis. If the two measurements differed by more than 5 mmHg, additional readings were taken.

Education level was classified into three categories: Diploma and less, Associate’s degree and Bachelor, Master and PhD. Family income, the total monthly income of all family members, was divided into four categories. The type of delivery included types of natural delivery and delivery by caesarean section. The job status, Cigarette smoking status, History of chronic illness, gestational diabetes and hypertension during pregnancy and the use of dietary supplement during pregnancy assessed at the beginning of the study. Sleep quality was evaluated by 18 items Pittsburgh Sleep Quality Index (PSQI) [42]. The Pittsburgh Sleep Quality Index (PSQI) were validated in Iranian population by Farrahi Moghaddam [43]. In a study conducted on pregnant women in Iran, the reliability of PSQI was reported 0.84 [42]. Physical activity status calculated by the Metabolic Equivalent (METs) [44–46]. The physical activity level was gathered by the international physical activity questionnaire (IPAQ) [45] and calculating metabolic equivalent of task [46]. The reliability and validity of this questionnaire had been assessed in the study by Rashidkhani et al [47].

Regarding the time of data collection, all questionnaires were filled in the first trimester of pregnancy before the 20th week of pregnancy, and in the field of EPDS, they were completed two months after delivery.

### Statistical analysis

In order to study the baseline characteristics, the people were divided into two groups, high PPD symptoms group, which included the mothers with score 13 and above, and low PPD symptoms group which included the mothers with score 12 and below. Frequency (percentage) was used to describe qualitative variables and mean (SD) was used for quantitative variables. In order to check the homogeneity of the distribution of variables in two groups, chi-square tests, Fisher’s exact test and two independent samples t-test were used. The dietary patterns were extracted using exploratory factor analysis (EFA) based on food groups. The results of sample size adequacy for factor analysis through the Kaiser-Meyer-Olkin (KMO) and Bartlett’s sphericity test reported. In EFA, the number of latent factors was determined using eigenvalues greater than one and also using scree plot. Rotation of factors through Varimax with Kaiser Normalization method was performed. The naming of the patterns was done based on the food items included in that factor.

To determine the relationship between the incidence of high PPD symptoms and food patterns, multiple logistic regression model was used. In this model, the effect of baseline characteristics such as subjective sleep quality, religion, job status, energy, waist circumference and BMI in the first trimester were controlled. Adjusted risk ratio (OR _adj_) and 95% confidence interval were calculated. All analyzes were performed in SPSS software version 24, by considering a significance level of 5%.

## Results

A total of 1028 women participated in the study included 247 high PPD symptoms individuals and 781 low PPD symptoms individuals. Based on depression scores, the overall presence of high PPD symptoms prevalence, which indicated the mothers whose depressive symptom scores were above 13, was 24%. The mean (SD) of age was 28.89(5.32) years. The description of baseline characteristics according to two groups, high PPD symptoms group which included the mothers with score 13 and above and low PPD symptoms group which included the mothers with score 12 and below, is presented in Table 2.

**Table 2:** Comparison of baseline characteristics of participant

Variables	levels	Groups				p
		low PPD symptoms		high PPD symptoms		
		N	%	N	%	
Level of education	Diploma and less	323	49.31	184	49.33	0.752*
	Associate’s degree and Bachelor	278	42.44	165	44.24	
	Master and PhD	54	8.24	24	6.43	
Level of income (IRR)	< 10,000,000	71	10.84	43	11.53	0.419*
	10,000,000–30,000,000	502	76.64	292	78.28	
	30,000,000–50,000,000	53	8.09	27	7.24	
	> 50,000,000	29	4.43	11	2.95	
Cigarette smoking	Yes	2	0.31	0	0.00	1**
	No	653	99.69	373	100.00	
Job status	Housewife	485	74.05	284	76.14	0.079*
	University Student	27	4.12	9	2.41	
	Employed	143	21.83	80	21.45	
History of chronic illness	No	482	73.59	269	72.12	0.935*
	Yes	173	26.41	104	27.88	
Hypertension in pregnancy	No	634	96.79	364	97.59	0.928*
	yes	21	3.21	9	2.41	
Gestational Diabetes	No	605	92.37	344	92.23	0.681*
	yes	50	7.63	29	7.77	
dietary supplement use during pregnancy	No	231	35.27	133	35.66	0.944*
	Yes	424	64.73	240	64.34	
Types of Childbirth	Not available	12	1.85	11	2.98	0.373*
	Vaginal	192	29.63	99	26.83	
	Natural	17	2.62	19	5.15	
	Epidural	14	2.16	1	0.27	
	Induction	120	18.52	68	18.43	
	Vacuum	7	1.08	4	1.08	
	Forceps	19	2.93	15	4.07	
	*cesarean*	267	41.20	152	41.19	
baby’s gender	Girl	316	48.32	185	49.73	0.87*
	Boy	338	51.68	187	50.27	
sleep quality	poor	324	41.5	123	49.8	0.022*
	good	457	58.5	124	50.2	
		**Mean**	**SD**	**Mean**	**SD**	
Energy intake		2546.15	32.63	2339.28	48.49	0.001***
Waist circumference in the first trimester		95.73	9.92	94.39	9.72	0.063***
Age		28.86	5.37	29.02	5.14	0.68***
Met.		855.42	44.74	841.26	92.98	0.882***
BMI in the first trimester		26.006	4.36	25.51	3.89	0.117***
HDL		62.38	14.04	61.86	10.61	0.534***
TG		128.58	45.79	126.84	49.39	0.61***
FBS		79.69	7.66	79.28	7.91	0.462***
SBP		99.74	8.34	99.02	7.58	0.226***
DBP		61.35	7.52	61.10	7.28	0.64***

*Chi square test; **Fisher’s exact test; ***Independent sample t test

According to Table 2, in terms of sleep quality, the majority of people reported a good level (58.5% in low PPD symptoms PPD vs.50.2 in high PPD symptoms). Regarding the level of education, the highest percentage was related to the diploma level and below, so that it was 49.23% in the high PPD symptoms group vs. 49.31% in the low PPD symptoms group. In terms of occupation, most people in the two groups were housewives (76.14 in the high PPD symptoms group vs. 74.05 in the low PPD symptoms group). Comparing the two groups based on income, smoking, history of chronic disease, gestational diabetes, dietary supplement use during pregnancy, types of Childbirth, and the gender of the baby did not show any significant difference between the two groups (p > 0.05). In terms of energy intake, in the low PPD symptoms vs. high PPD symptoms groups, it was equal to 2546.15 kcal per day and 2339.28 kcal per day, respectively (p = 0.001). In terms of systolic and diastolic blood pressure, waist circumference, HDL, TG, FBS, age and met equivalent both groups were homogeneous (p > 0.05).

The results of EFA in determining the later food patterns extracted four patterns. The factor loading matrix is shown in Table 3. These four patterns explained 34% of the variance, namely ‘prudent pattern’, ‘sweet and dessert pattern’, ‘junk food pattern’ and ‘western pattern’. After evaluating the eigenvalues, the scree plot test and interpretability, factors with eigenvalues ≥ 1·0 were retained, and individual food items with absolute factor loadings ≥ 0·25 were considered to contribute significantly to the pattern [48]. the detailed foods of the four dietary patterns mentioned above are: prudent pattern (high intake of vegetables, fruit and juice, nuts and beans, low-fat dairy products, liquid oil, olive, eggs, fish, whole grains), sweet and dessert pattern (high intake of tea, sweet jam and desserts and refined grain), junk food pattern (high intake of salt, salty snacks and solid oil) and western pattern (high intake of red meat and processed meats and organs). The overall Cronbach’s Alpha was0.74 and Cronbach’s alpha of each food group was as follows: vegetables 0.85, fruit and juice0.86, nuts and beans 0.67, low-fat dairy Products0.85, liquid oil 0.65, olive 0.75, eggs 0.72, fish 0.71, whole grains 0.83, tea 0,72, sweet jam and desserts 0.64, refined grain 0.61, solid oil 0.82, salty snacks 0.8, salt 0.65, red meat 0.84, processed meats and organs 0.73, high-fat dairy 0.74, industrial drinks 0.7, chickens 0.68.

**Table 3:** Rotated component matrix of major dietary patterns identified in women

Food Groups	Component			
	prudent pattern	sweet and dessert pattern	junk food pattern	western pattern
Vegetables	0.707			
Fruit and Juice	0.697			
Nuts and Beans	0.488			
Low-Fat Dairy Products	0.482			
Liquid Oil	0.440			
Olive	0.352			
Eggs	0.301			
Fish	0.261			
Whole Grains	0.258			
Tea		0.732		
Sweet Jam and Desserts		0.684		
Refined Grain		0.346		
Solid Oil			0.691	
Salty Snacks			0.513	
Salt			0.384	
Red Meat				0.663
Processed Meats and Organs				0.494
High-Fat Dairy				0.488
Industrial Drinks				0.363
Chickens				− 0.258
variance explained percentage	13.3	7.9	6.7	5.9
Extraction Method: Principal Component Analysis. Rotation Method: Varimax With Kaiser Normalization.				
Kaiser-Meyer-Olkin measure of sampling adequacy = 0.67; Bartlett’s test of sphericity < 0.001				
Total Cronbach’s Alpha = 0.74				

**Notes:** Absolute values less than 0.2 are not displayed for simplicity. Numbers in bold indicated main food groups with significant correlation (> 0.25) within each dietary pattern

For investigating the relationship between high PPD symptoms incidence and dietary patterns, four dietary patterns which extracted by EFA were enter into logistic regression model. Also, multiple logistic regression results were adjusted for effects of the other variables (Table 4).

**Table 4:** Results of Multiple logistic regression in relation of high PPD symptoms and Dietary pattern

Dietary pattern	Model	T1	T2	T3	P
		OR (95% CI)	OR (95% CI)	OR (95% CI)	
Prudent Pattern	#1	1(Reference)	0.50(0.36–0.72)	0.46(0.32–0.65)	< 0.001
	#2	1(Reference)	0.52(0.37–0.74)	0.52(0.36–0.75)	< 0.001
	#3	1(Reference)	0.53(0.37–0.76)	0.55(0.37–0.85)	< 0.001
	#4	1(Reference)	0.53(0.37–0.75)	0.55(0.37–0.85)	< 0.001
Sweet and Dessert Pattern	#1	1(Reference)	1.06(0.75–1.50)	0.95(0.66–1.35)	0.517
	#2	1(Reference)	1.15(0.81–1.65)	1.19(0.81–1.74)	0.428
	#3	1(Reference)	1.13(0.79–1.60)	1.14(0.78–1.68)	0.495
	#4	1(Reference)	1.11(0.78–1.59)	1.13(0.77–1.66)	0.548
Junk Food Pattern	#1	1(Reference)	0.99(0.70–1.41)	1.06(0.75–1.51)	0.721
	#2	1(Reference)	0.97(0.68–1.38)	1.23(0.86–1.77)	0.865
	#3	1(Reference)	0.98(0.69–1.41)	1.21(0.84–1.74)	0.943
	#4	1(Reference)	0.99(0.69–1.42)	1.17(0.81–1.69)	0.402
Western Pattern	#1	1(Reference)	1.56(1.08–2.25)	1.78(1.24–2.56)	< 0.001
	#2	1(Reference)	1.70(1.17–2.47)	2.54(1.71–3.78)	< 0.001
	#3	1(Reference)	1.81(1.24–2.66)	2.73(1.82–4.10)	< 0.001
	#4	1(Reference)	1.79(1.22–2.63)	2.67(1.78–4.02)	< 0.001

• #1 Univariate logistic regression

• #2: Multiple logistic regression, adjusted by energy intake and age

• #3: Multiple logistic regression, adjusted by the effect of all variables except sleep quality

• #4: Multiple logistic regression, adjusted by the effect of all variables

• T: tertiles of dietary patterns, T1:tertile1, T2:tertile2, T3:tertile3

• All variables means energy intake, age, waist circumference, BMI in the first trimester, Types of Childbirth, Job status, Religion, level of income, babys gender, physical activity, and sleep quality)

The results of multiple logistic regression showed in the prudent pattern, which included healthy foods and fruits, a high adherence to the healthy foods and fruits was associated with a lower risk of high PPD symptoms than a low adherence, so that in the model #4 with controlling all confounding variables, the risk of high PPD symptoms in the second tertile was 47% less than the first and the third tertile was 45% less than the first tertile (OR_2vs1_ = 0.53, OR_3vs1_ = 0.55 P < 0.05), there were similar results in other models.

There was no significant relationship between adhering to the Sweet and Dessert Pattern and Junk Food Pattern with incidence of high PPD symptoms (p > 0.05). In the Western pattern which included high intake of red and processed meat, a high adherence to this pattern was associated with a higher risk of high PPD symptoms than a low adherence, so that in the model #4 with controlling all confounding variables, mothers who were in the third tertile had 2.67 times more risk to getting high PPD symptoms than mothers in the first tertile (OR = 2.67, P < 0.001) and mothers who were in the second tertile had a 1.79 times higher risk of catching high PPD symptoms PPD than mothers in the first tertile (OR = 1.79, P = 0.002). There were similar results in other models.

## Discussion

PPD is a major depressive disorder that mainly begins within one month after delivery [49]. This disease as a fundamental issue in public health not only affects the life of the mother, but also has an effect on the growth and development of the child, cognitive, social and behavioral opinion [10]. It can even lead to mother’s suicide and harm to the child. So, the present study aimed to determine the relationship between dietary patterns and the occurrence of high PPD symptoms in women participating in the first phase of Iranian Maternal and Child Health cohort study, Yazd, Iran.

Our results showed the incidence of high PPD symptoms, which indicated the mothers whose depressive symptom scores were above 13, was acquired at 24%. In similar studies in Iran [50–53], the prevalence of PPD was estimated 15–41%. In the present study, Examining the homogeneity of the distribution of quantitative and qualitative variables in high PPD symptoms vs. low PPD symptoms groups showed that only the energy intake variable was different in the two groups, so that energy intake was significantly higher in low PPD symptoms women. In line with the present study, in the study of Most et al., 2020, it was emphasized that women with PPD consumed less energy and have weight lost compared to the period of pregnancy [15]. According to research, about 50–75% of depressed patients also have anorexia. Anorexia aggravates physical and mental problems over time, and if treatment is not done, it causes very dangerous consequences for patients. Therefore, it seems logical that women with PPD have less energy consumption and lose weight.

Sleep quality is another concern of women after childbirth. Sleep disturbance can occur in the form of deprivation, which refers to a lack of sleep, or fragmentation, which refers to numerous awakenings after an individual falls asleep [54]. Sleep is a basic physiological need and a complex physiological process that restores physical agility and energy. The main cause of sleep disturbance after delivery is infant signaling during the night [55]. A sleep quality study at postpartum of women suggested that poor sleep quality is linked with increased depression and anxiety symptoms in women at six months after delivery. Moreover, the study was used both the Pittsburgh Sleep Quality Index (PSQI) to investigate the relationship between changes in self-reported sleep patterns and depressive symptoms, revealed that those who are at a high risk of PPD are also at a higher risk of developing depressive symptoms later in their postpartum period if sleep issues persist or improve very minimally over time [56]. While these studies produced insightful results regarding the relationship between sleep disturbance and the risk of developing PPD, there was heterogeneity in terms of the sample characteristics and the timepoints at which PPD symptoms were studied. In this study, although poor sleep quality amount in high PPD symptoms group were approximately 8% more than low PPD symptoms group (p = 0.022) that shown in Table 2, but after adjustment of sleep quality, no significant difference between dietary patterns and high PPD symptoms was observed (Table 4).

The results of EFA confirmed the extraction of four patterns were named based on the food groups included in them. For instance, prudent pattern included high intake of healthy foods, fruits and vegetables. Results of multiple logistic regression showed, compare to low adherence, a high adherence to the healthy foods and fruits was associated with a lower risk of high PPD symptoms. In line with our study, a study conducted by Maracy et al. in 2014 in Iran, showed a significant relationship between the incidence of PPD and the dietary pattern of fruits and vegetables, so that women who consumed more fruits and vegetables during pregnancy had a lower odds of getting PPD [30]. They also emphasized that this pattern has a protective effect against PPD that it was consistent with our study. Contrary to the results of our study, a study in Japan showed that there was no significant relationship between dietary pattern and PPD [48]. A cohort study conducted by Baskin et al. in 2017 showed that there was no relationship between dietary patterns and incidence of PPD in pregnant women [57]. In general, considering the different conditions of the studies and attention to the existing contradictions, it seems that more studies are needed to determine the role of dietary patterns in the incidence of depression.

Previous studies concentrating on single food groups have shown that higher intakes of fish [58], fruit and vegetables [59] and fiber intake [60] are associated with lower depressive symptoms. However, analyzing food groups individually has limitations because the role of these individual components is examined without considering the complication of a dietary pattern [61]. An examination of food groups showed that higher vegetable intake was related to lower depression and this association was as expected. Thus, higher consumption of non-refined grains, vegetables, fruit, potatoes, fish and olive oil were inversely related to depression or lower odds of a current diagnosis, while higher consumption of poultry and high fat dairy products was positively associated with higher depressive symptoms [62]. Contrary to a meta-analysis indicated that high-fish consumption reduced the risk of depressive symptoms [58], Gibson, et al. found no associations between fish and depressive symptoms that lack of findings could be due to the generally low levels of fish consumption in that society [62].

Another posteriori pattern which extracted by EFA in this study was the sweet and dessert pattern, it was related to the consumption of sweets, desserts and refined grains. The findings of our study showed that there was no significant relationship between adhering to the Sweet and Dessert Pattern and PPD incidence. Many studies pointed out a significant effect of the sweet and dessert pattern on depression so that, a high adherence to this pattern was associated with a higher odds ratio of PPD than a low adherence [57, 63, 64]. According to the contradictory results of previous studies, it seems that longitudinal studies should be planned in this field.

The third posterior dietary pattern extracted by EFA was junk food pattern. This pattern focused on salty snacks and solid oils. The results of our study showed that there was no significant relationship between adhering to this pattern and incidence of PPD symptoms. Some studies showed the negative effect of unhealthy diet such as salty snacks on depression so that a high adherence to this pattern was associated with a higher odds ratio of PPD than a low adherence [65–67]. In the present study, although the adherence to this pattern had not significant relationship with high PPD symptoms occurrence, a high adherence to this pattern was associated with a higher risk of high PPD symptoms than a low adherence. The non-significance and inconsistency of our findings with previous studies can be due to the study design and different sample size. On the other hand, the present study was the first phase of a cohort study, maybe different results will be obtained in the next follow-ups. Results from previous mental health focused papers showed that vegetables, fruit, high fiber, fish, low fat dairy were associated with depression inversely, and sugar-sweetened beverages, fast food, snacks and sweets were positively associated with depressive symptoms. [68–71].

The fourth extracted posterior Dietary pattern was western pattern. This pattern focused on processed and red meat and organs consumption. Results of our study showed that a high adherence to the processed and red meat was associated with a higher risk of high PPD symptoms than a low adherence. In line with the present study, Nucci et al., in a systematic review and meta-analysis with a review of 18 studies and a population of 160,000 people, concluded that consumption of red meat and processed meat increases the chance of depression [72]. Therefore, according to the importance of the mentioned review, and the findings of the present study, paying attention to the western diet and trying to reduce adherence to it in pregnant women can help to a large extent in preventing the development of high PPD symptoms in women.

The malefic effect of Western pattern on inflammation and coronary heart disease [73, 74] can play a role in the pathogenesis of depression [75, 76]. However, further studies are needed to research the association between western pattern, the inflammation process and depression.

There are several plausible mechanisms underlying the association between the dietary patterns and depression are complicated and reasons for bilateral relations can be provided. First, changes in appetite and weight, poor motivation, and low energy levels are symptoms usually seen in depressed people [77], for this reason, changes in energy intake and reduction in personal health behaviors occur [78]. Because unhealthy foods usually are prepared faster and easier than healthy foods, it could be expected that the diet quality may decrease. Second, deficiencies in certain vitamins [79], minerals [80], and essential fatty acids [81] may affect depression by directly influencing biological pathways related to the pathophysiology of depression. Vegetables are an important source of minerals, fiber, alpha-linolenic acid, vitamins, and other anti-oxidants. The high level of antioxidants in fruits and vegetables could counteract free radicals and oxidative stress, which has been shown to be increased in depressed persons [82–84]. Also, a high content of folate in some leafy green vegetables and legumes has a positive role in improvement of depression diseases [85]. It has been suggested that low levels of folate might increase the risk of depression and result in reduced availability of S-adenosylmethionine, which can result in disruption of formation of myelin, neurotransmitters and membrane phospholipids [86]. Low levels of folic acid and zinc, whose found in vegetables and non-refined grain products, have been associated with depression [87, 88]. The other studies suggested the protective effect of long-chain ω-3 polyunsaturated fatty acids in depression. These are a major component of neuron membranes that have anti-inflammatory properties [76, 89–91]. Third, diet may indirectly affect depression through negatively affecting the gut microbiome and causing low level inflammation, which leads to the risk of depression. Finally, we should consider that the association between dietary pattern and depression may have been confounded by social economic status and income [92, 93]. Many of the food groups associated with lower depression are typically more expensive and more often consumed by those of higher income, SES and education, and although we adjusted for education level, there may have been residual confounding by social economic status [94]. Some limitations of our study should be noted. First, the local and cross-sectional nature of the study, which might limit the capability to communalize the results to other areas. Second, is the memory bias that may occur when a self-reported and long-term dietary assessment method such as the semi quantitative FFQ is applied. Third, the instrument of PPD in our study was EPDS questionnaire which evaluated the symptoms of depression and it is not a diagnostic tool, in this study PPD was not diagnosed by psychiatrists and high depressive symptoms at 2 months postpartum were considered high PPD symptoms; however, definitions may vary. Forth, in this study, from an initial sample of 3000 subjects only 1028 women concluded the research.

One of the strengths of this study is the large sample size and the use of cohort data. Another strength of the study is the adjustment of some confounder variables like sleep quality, which can make an impact on the results.

## Conclusion

The results of this study showed that high adhering to the Western Pattern increased the risk of high PPD symptoms. On the other hand, Sweet and desert, junk food pattern had no significant relationship with high PPD symptoms. Therefore, it is suggested that the women health care providers have a particular emphasis on the adherence to healthy food patterns such as the prudent patterns.

## Data Availability

The data that support the findings of this study are available from the corresponding author upon reasonable request.

## Abbreviations

PPD: Postpartum Depression
FFQ: Food Frequency Questionnaire
EPDS: Edinburgh Postnatal Depression Scale
EFA: Exploratory factor analysis
MLR: Multiple logistic regression
MSQ: Mental status questionnaire
WC: Waist circumference
SBP: Systolic blood pressure
DBP: Diastolic blood pressure
PSQI: Pittsburgh Sleep Quality Index
METs: Metabolic Equivalent
HDL: High density lipoprotein
TG: Triglyceride
FBS: Fasting blood sugar
